# How greedy is too greedy? A network toy model for evaluating the sustainability of biased evolutionary dynamics

**DOI:** 10.1098/rstb.2022.0260

**Published:** 2024-01-01

**Authors:** V. P. Weinberger, N. Zalaquett, S. Abades

**Affiliations:** ^1^ Center for Resilience, Adaptation and Mitigation (CReAM), Universidad Mayor, Temuco, 4801043, Chile; ^2^ PLR Physics Ludique Research, Santiago, 9761013, Chile; ^3^ GEMA Center for Genomics, Ecology & Environment, Universidad Mayor, Santiago, 8580745, Chile

**Keywords:** socio-ecological systems, network toy-model, energy sequestration, sustainability, socio-metabolism, energy-based flowing networks

## Abstract

Modern humanity has changed the biosphere at a global scale, threatening its own sustainability. It is claimed that through technology humans maximize the extraction of energy from the natural system towards their own benefit, with rates of appropriation that surpass the time-scales for systemic adaptation. This time-decoupled coevolutionary dynamic is at the core of human societal unsustainability. Here, we developed *in silico* experiments of an open energy-based flowing network toy model of natural systems and study the effects that *greedy* evolutionary strategies, resembling human societal demands, have upon the performance and *scarcity tolerance* of the system. We aim to determine the flexibility that those biased evolutionary dynamics have for matching or surpassing natural evolution outcomes. We studied four different indexes of system’s growth and development (total system throughflow (TST), average mutual information, ascendency and entropy difference) and compare their scarcity tolerance and performance outcomes with respect to four different greedy scenarios. The results showed that greedy strategies rarely surpassed the tolerance and performance achieved by natural systemic evolution. The nature of the greedy scenarios developed were closely related to increases in TST and therefore, we emphasized this comparison. Here, the maximum percentage of greedy networks capable of surpassing natural dynamics was around one-third (approx. 35%). However, results suggest the existence of a space parameter where local increases of energy flow can outperform the outcomes of natural systemic evolution, but no evident network property seems to characterize those greedy networks. A mild inverse relationship was found between the number of links that greedy nodes have towards the output and their capacity to outpass the control evolution. As many of the human societal effect upon biospheric processes have dissipative byproducts, knowing that such dynamics might diminish the systems tolerance and performance suggest care in their (ab)use.

This article is part of the theme issue ‘Evolution and sustainability: gathering the strands for an Anthropocene synthesis’.

## 1. Introduction

The living system of the Earth emerges from complex relationships of energy transformation processes that have evolved through time into the intricate inter-relationships that now compose the biosphere, and continues to coevolve as a planetary force with the natural system [1–3]. It was within this intricate network of relationships that the human species, as part of the living system, appeared in some time event during its evolution and powered the construction of greater human societal scales at greater population numbers [4–7]. However, modern humanity has now changed the biosphere at global scale, driving a new geological epoch, i.e. the Anthropocene [8]. This new epoch characterizes for threatening the natural system’s conditions in which our species has thrived, and with this, our own sustainability [9–12]. Understanding the relationships that human activities and demands have within the natural system has then become key for determining the sustainability of the coupled system, and required for properly assessing human limits and/or opportunities for growth [4,13].

There is ample concern in socio-ecological theory about the processes and mechanisms that have allowed such great expansions of human societies, as no single species appears capable of modifying natural relationships on its own at the global scale, and even less at rates faster than the evolutionary time-scales [4,6,14,15]. It is claimed that through sociocultural niche construction (*sensu* [4], see also Ellis [16]), humans have developed technological strategies for maximizing the extraction of energy from the natural system towards their own benefit ([5,15,17–19]; see also [20]). These strategies are supposed to be selected by natural selection, a hypothesis usually known as ‘maximum power principle’ in ecology [21,22]. However, as rates of techno-cultural evolution of the human societies can occur much faster than regular evolutionary time-scales [23], those sequestration dynamics surpass the rates at which the whole system can respond and adapt. This time-decoupled coevolutionary dynamic is claimed as the core of human societal unsustainability [19].

Are natural systems capable of sustaining the rapid evolution of those *greedy* strategies? How are system’s dynamics affected by the evolution of those *biased* configurations? Here, based on systemic approaches of natural systems [13,24], we developed a network toy model for studying such claims. Through simulations (open code at github: https://github.com/vanewi/flownet_evolution) and theory (see the electronic supplementary material, SI), we developed different *in silico* experiments for comparing the evolution of systemic versus biased evolutionary strategies. We study the effects that energy appropriation at specific network structures that do not necessarily comply to systemic dynamics (resembling greedy strategies) have upon system’s scarcity tolerance and performance. We want to determine the diversity and frequency of greedy strategies that do not compromise the sustainability of the whole system. This analysis could help understand the flexibility that modern human societies have for re-integrating back into regular natural dynamics and achieve sustainability.

## 2. Framework

We start assuming natural systems as open systems that continually exchange energy with the environment and organize into structured relationships that sustain dynamics of order within its boundaries [1,2,25–27]. In these models, the system receives energy from external inputs and continually uses and transforms it through different processes, before finally dissipating that energy back to the environment. These processes of energy transformations are conservative, meaning that no energy is created or lost within the natural system’s boundaries; in other words, all incoming energy is either transformed into new energy forms (i.e. changed to new biochemical forms) or dissipated.

Network theory can be used for describing such energy relationships [28–30] and has shown useful for detecting recurrent patterns shared across different ecological organization, such as trends of system’s growth and development (i.e. system’s evolution) [31–33]. Energy-based flowing networks have been amply used for representing ecosystem dynamics [25,32,34]. Here, the energy from the sun and/or geothermal chemistry from the Earth's crust serves as the external energy input, which is used and transformed by biological processes evolved from the metabolisms of organisms, comprising the ecosystems’ dynamic. Usual representations group these biological transformation processes at different ecological organization scales, from chemical reactions (e.g. metabolism, [35]), functional ecological groups (e.g. producers, consumers, [25]), species (e.g. trophic networks [32]) and the biosphere [27]. Within this framework, system’s growth is usually defined as greater energy flow within system’s boundaries, maximizing its power, either accumulating it in its components and/or in the fluxes among them [22,32]. On the other hand, system’s development relates to increases in the organization and/or complexity in the system, building on structures that show signatures of persistence and/or efficiency [24,32,36]. Both processes are assumed to describe system’s evolution [22,36], and have been described from properties of networks, with different systemic indexes proposed for their quantification (see box 1) [29,32,37].

From a systemic perspective, recycling dynamics or *cycles* appear as a key mechanism for systemic growth and development [31,42,43]. This network structure increases the length paths of energy flow within the system, developing positive feedbacks between its constituent components that allows greater appropriation dynamics [40,42,44]. Cycles also give the system some autonomy from boundary conditions [31,43,45]. Moreover, because of their combinatorial arrangement in networks, they can greatly increase system’s complexity, even at small network dimensions (e.g. networks of 10 nodes can accommodate 1 112 073 different directed cycles if fully connected). Hence, the cycling dynamics observed in relational networks can serve as a valuable tool for interpreting mechanisms of energy appropriation within a system, enabling the identification of configurations that promote the maximization of its systemic growth and development. We use recycling dynamics with that aim in this research.

### (a) Toy model: definition, dynamics and scarcity tolerance of flowing connected directed networks (FCDN)

We start considering natural systems as a graph *G* ≡ *G*(N, E), where N is a set of nodes *n*_*i*_ (with *i* = 1, …, *N*, *N* + 1, *N* + 2) representing energy forms or states, and E is a set of directed edges or links *e*_*ij*_ representing energy transformation processes from form or state *n*_*i*_ into form or state *n*_*j*_. For instance, for the generic representation of an ecosystem, the nodes associated with the input source correspond to energy forms entering the natural system (e.g. electromagnetic radiation), which are used and transformed by biological process (e.g. photosynthesis, corresponding to graph’s links), giving rise to new biochemical energy forms (e.g. glucose, starch, cellulose, etc. representing possible nodes in the graph). These energy forms can be further used by other biological processes (e.g. catabolic reactions of organisms’ metabolism), giving rise to other energy forms (e.g. aminoacids, proteins, fats, etc.). Between these transformation processes, energy can also dissipate out of the system (e.g. heat production).

**Figure 1.**
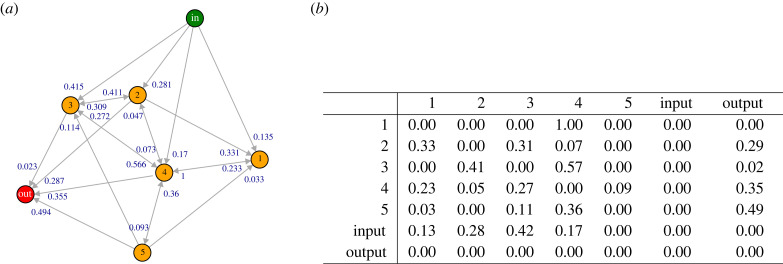
Flowing connected directed network (FCDN) representations. (*a*) Network representation of a natural system, with one input (green), one output (red) and five core nodes (yellow) *n*_1..5_, containing 13 cycles. Link’s weights are written next to arrow’s head for clarity (*b*) Adjacency matrix (A) of FCDN represented in figure 1*a*.

Our objective in this study is not to delineate and depict the comprehensive set of actual energy transformation pathways inherent to natural systems—that is, the entirety of energy forms and processes necessary for representing ecosystem dynamics. We acknowledge the immense complexity and probable incompleteness of our understanding of such a representation on a full scale. Consequently, we have chosen to explore potential energy flow configurations via simulation methodologies. This strategy enables the illustration of various plausible energetic flow configurations within natural systems, and the impacts of different evolutionary dynamics on their systemic behaviour. To achieve this, we will apply certain constraints to the general set of *G* to encapsulate our definition of natural systems, referred as *flowing connected directed networks* (FCDN’s; see figure 1*a*).

For this, consider matrix A≡A(G) as the adjacency matrix of *G*, with element $A_{ij}$ the share of energy that flows from form *n*_*i*_ into form *n*_*j*_ through the $A_{ij}$ energy transformation process. Energy share is normalized by output ($\sum_{\;j}A_{ij}=1$), meaning that element $A_{ij}$ is actually the proportion of total energy that fluxes out of node *n*_*i*_ into node *n*_*j*_; therefore, matrix A is *row-normalized* and all their weights are between [0, 1]. Self-loops in A are not allowed (i.e. $A_{ii}=0$); that is, energy is always *flowing* in our networks. Any node *n*_*j*_ for which $\sum_{i}A_{ij}=0$ is defined as an ‘input node’ *n*_*N*+1_. Nodes *n*_*i*_ with $\sum_{\;j}A_{ij}=0$, will be referred as an ‘output node’ *n*_*N*+2_. All the other nodes *n*_1···*N*_ will be referred as ‘core nodes’. For any representation A of our networks, there will be a single input node (*In* = *n*_*N*+1_), a single output node (*O* = *n*_*N*+2_) and *N* core nodes *n*_*i*_ with *i* = (1 · · · *N*). There is no loss in generality as, if there are more inputs, we could for most purposes reduce them to a single node, adjusting the share weights. The same works for output. Every core node *n*_*i*_ needs to be either directly or indirectly connected to both the input node *"*in*"* and the output node "out*"*. For exemplification of FCDN’s adjacency matrices, see figure 1*b*.

The network topologies just described as FCDN’s can reach analytical tractable steady states of energy flow per node ($X_{i}^{*}$) for all 0 ≤ *i* ≤ *N* core elements if supplied with a constant input (see the electronic supplementary material for details). This steady-state condition also allows us to obtain the stable flux of energy through all network’s edges as $T_{ij}=X_{i}^{*}A_{ij}$, with *A* the core adjacency matrix $A_{ij}=A_{ij}$ with i,j = (1 · · · N). Moreover, FCDN’s also show analytical tractable pulsating dynamics (detailed in the electronic supplementary material), or dynamics in which the input operates only at frequency *p* (the input ‘misses’ *p* iterations).

Given FCDN’s pulsating dynamics, we focus on the *energy flux decay rate* (*b*) of accumulated energy in the system under sudden scenarios of no input (i.e. scarcity dynamics; see the electronic supplementary material for details). Scarcity dynamics can be understood as natural and/or punctual environmental disturbances that affects the entrance of energy in the system, altering its dynamic. Such could be the case of regular seasons, with winter affecting the energy that enters an ecosystem, or a volcano explosion or an eutrophication event within the system, all of them situations that preclude the entrance of solar radiation (e.g. energy) into the natural system. In extreme scenarios (when the energy input is completely precluded), there is a sudden change of the only possible fixed point of the system, from the steady state to its death (given its property as a dissipative system). Therefore, FCDN’s that can sustain the energy within the system’s boundaries for extended periods also potentially delay their demise. This index can be viewed as a measure of a system’s *scarcity tolerance*, and relates to positive and desirable attributes for FCDN’s.

The energy flux decay rate *b* is directly related to the maximal eigenvalue of the core adjacency matrix *A*, as *b* = *E*_max(*A*)_ − 1. However, notice that the maximal eigenvalue also measures the rate at which the system returns to its equilibrium under a perturbation, regularly conceived as the system’s resilience and is determined as *R* = 1 − *E*_max(*A*)_ ≡ −*b* [46,47]. This inverse relationship between indexes is tricky: for one hand, under scarcity dynamics, greater resilience *R* means that the system will more rapidly loose its accumulated energy—a property we just called as undesirable for FCDN’s, as it suggests faster trajectories to possible death. This is contrary to the expected behaviour of tolerant networks. On the other hand, whenever the input dynamic is restored and the system might be able to return to the previous equilibrium (considering death is avoided), more resilient systems should recover faster than systems with greater tolerance. In this research, we focus on system’s capabilities for maintaining energy flowing within its borders, and therefore, we will not discuss the resilience metric, and will only focus FCDN’s scarcity tolerance *b*. In other words, and based on the former examples, we will focus on a system’s capacity to maintain its energy flow the longest during events such as winter, eruptions, eutrophications, etc., as a possible mean to recover its regular properties if the energy dynamic is eventually recovered (e.g. spring arises, ashes go away and/or the algae clears).

**Box 1. Systemic indexes in relational networks.**

Network theory has been amply used for studying the properties of natural systems, with great development in ecosystem theory [28,37]. Some systemic indexes that exist for studying system’s growth and development and that are used in this research are as follows.

1. *Total system throughflow* (TST): in balanced networks (when the total energy entering a node *n*_*i*_ is the same as the energy leaving that component), the TST is defined as the sum of all the fluxes ($\sum_{i=1}^{N}\sum_{\;j=1}^{N}T_{ij}$) of energy that flow through network’s components *n*_*i*_ with *i* = 1 · · · *N*. This metric is intimately associated with the activity of the system, and the greater it is, the more ‘power’ the system uses. Some theories claim that natural systems evolve towards greater power or TST [21,22,27].
2. *Average mutual information* (AMI): defined as an information based index, AMI quantifies the average information generated from pairwise interactions between network’s components. The index considers the joint probability of the flow *T*_*ij*_/TST, the marginal probability of that specific flow to occur towards component *j* $\sum_{i}T_{ij}/$TST, and the conditional probability that the flow passes through component *i* $T_{ij}/\sum_{\;j}T_{\;ji}$. It is finally defined as AMI = $\sum_{i,j=1}^{N}(T_{ij}/\mathrm{TST})\log(T_{ij}\mathrm{TST}/\sum_{i}T_{ij}\sum_{\;j}T_{ij})$, and quantifies the development of the system, with more developed systems having greater AMI values [32,38].
3. *Ascendency* (ASC): this information-based index scales the average mutual information of flows in the system (i.e. AMI) by its total throughflow, as $ASC=\sum_{i,j=1}^{N}T_{ij}\;\log(T_{ij}\mathrm{TST}/\sum_{i}T_{ij}\sum_{\;j}T_{ij})$, and is used as an index for quantifying both system’s growth and development [32,39].
4. *Entropy difference* (EDiff): inspired by the common consideration that living systems are capable of sustaining a low entropy within their borders by exporting entropy to the surroundings [1], this proposed index quantifies the difference in entropy between the energy input versus its output from the system. The input entropy is determined by the entropy in throughflow attained by all the core nodes that are directly connected to the input node in. Let us first define ${\mathrm{TST}}_{\mathrm{in}}=\sum_{i\in \mathrm{in}}\sum_{\;j}^{N+2}T_{ij}\equiv \sum_{i\in \mathrm{in}}T_{i}$. Now we define our input entropy as $S_{\mathrm{in}}=-\sum_{i\in \mathrm{in}}(T_{i}/{\mathrm{TST}}_{\mathrm{in}})\log_{2}(T_{i}/{\mathrm{TST}}_{\mathrm{in}})$. The output entropy is determined by the outflow attained by all the core nodes that are connected to the output node "out". Defining ${\mathrm{TST}}_{\mathrm{out}}=\sum_{i\in \mathrm{out}}T_{i(N+2)}$ we can express our output entropy as: $S_{\mathrm{out}}=-\sum_{i\in \mathrm{out}}(T_{i(N+2)}/{\mathrm{TST}}_{\mathrm{out}})\log_{2}(T_{i(N+2)}/TST_{\mathrm{out}})$.
5. *Finn index* (*Finn*): in flow analyses, this index is defined as a metric for quantifying the fraction of medium (energy in this case) that cycles within the system [40,41]. Based on the Leontief matrix $L={\left(I-A\right)}^{-1}$ for quantifying all indirect fluxes within the network, the index uses the diagonal elements for determining the total flow that returns to each node as $\mathrm{Finn}=\sum_{i=1}^{N}\sum_{\;j=1}^{N}(T_{\;ji}/\mathrm{TST})((L_{ii}-1)/L_{ii})$. In this research, we also consider the Finn Index per node *n*_*i*_: ${\mathrm{Finn}}_{i}=\sum_{\;j=1}^{N}(T_{\;ji}/\mathrm{TST})((L_{ii}-1)/L_{ii})$ as a local index that determines the amount of energy that cycles through that specific node.
6. *Energy flux decay rate* (*b*): once FCDN’s at steady-state are left with no energy input, core nodes start losing their energy flow and approaches the new system’s death equilibrium at an exponential rate. This index is directly related to the maximal eigenvalue of the core adjacency matrix A, as $b=E_{\max(A)}-1$ (details in the electronic supplementary material). The lower it becomes, the less tolerant the network is to scarcity (the decay rate is faster).

### (b) Evolutionary dynamics: mutation and selection algorithms

Different systemic goal functions, also known as *orientors* (*sensu* [48]), have been proposed as systemic indexes of open system’s natural evolution. These indexes emphasize increases in system’s growth, development or both (box 1), and are claimed to be maximized during system’s evolution. Analyses of their complementarity show that most can relate to key network properties [28,29,37,49], and because they also base on energy dynamics, they can be used for representing the evolution of our FCDN’s. However, a consensus around which index better describes the behaviour of evolving natural systems is still lacking. Our aim in this research is not to clarify such consensus, but to apply these indexes for the toy model evolution and use them for comparing expected systemic evolution of natural systems to greedy evolutionary scenarios that deviate from systemic goals.

The systemic goal functions evaluated for our toy model were (box 1): (i) the total throughflow in the network (TST), which measures the power achieved by a the system (hypothesized to increase in time) [21,22,27]; (ii) the network’s average mutual information (AMI), proposed for quantifying system’s development [32,38]; (iii) the network’s ascendency (ASC), which stands as an index for quantifying both system’s growth and development [32]; and a new proposed index for (iv) entropy difference between input and export flows (EDiff), which seeks quantifying the exported entropy by the system. Some of these metrics are claimed to be fundamentally related to the presence of cycles and recycling dynamics [32,43], but difficulties for directly performing cycle statistical analyses on networks with a relatively large number of nodes and links have hindered those tests. We correlated these indexes between each other, and with system’s cycling (Finn) and scarcity tolerance (*b*) (see the electronic supplementary material).

We start all simulations by initializing random adjacency matrices A, which are subsequently adjusted using algorithms to ensure their condition as FCDN’s. Our next step is to submit these networks ensembles to the evolutionary dynamic, subjecting them to recurrent mutations that can restructure their topologies (but their condition as FCDN is ensured at every step). Mutations can, for each network and within one iteration: (i) change the weight of a link (either in the vicinity of its former value or not), (ii) eliminate a link, or (iii) create new weighted links. We only consider mutations in the relationships among biological transformation processes, therefore, only edges between the core nodes can mutate. Input links are never changed, while output links may only change their weight. Mutations represent stochastic changes at the local scale (e.g. the creation, elimination or changes in shares of energy transformation processes), introducing local variability in the system with potential systemic effects. This variability can be selected, giving rise to the evolutionary dynamic.

Based on the mutation variability created per iteration, selection algorithms: (i) detect whether the new mutated configuration complies with a specified condition, that is, the maximization of the desired goal function or orientor, (ii) if optimized, fix that mutation as an ‘event’ in the ‘evolutionary history’ of the evolved network from the ensemble, and (iii) submit this new fixed mutated network configuration to further mutations. The algorithms repeat until evolutionary iterations of the experiment are finished. These algorithmic approaches are common in ecosystem modelling, usually referred to as *structural dynamic modelling* (*sensu* [50]).

#### (i) Large and short-term evolutionary dynamics

**Figure 2.**
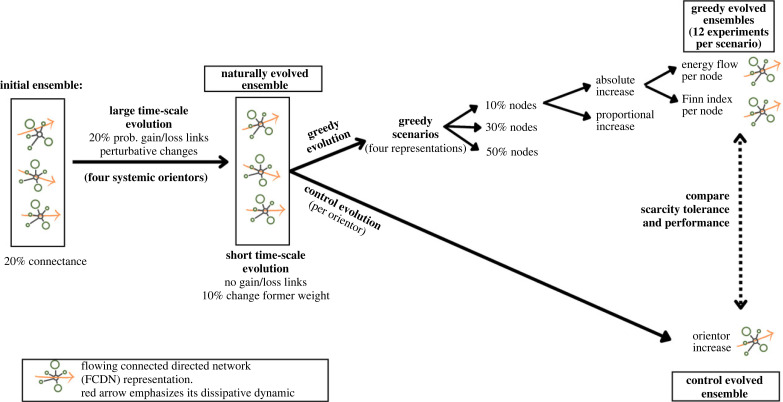
Experimental design for the simulated experiments. For space clarity, only one branch of the experimental design is expanded, and the rest of the branches follow the same expanded logic. Therefore, arrows for the 30% and 50% nodes are omitted, and are identical to the 10% node expansion.

Simulations submitted network ensembles of FCDN’s to two different mutation algorithms (figure 2), corresponding to (i) large time-scale and (ii) short time**-**scale evolutionary processes. The goal of such differentiation relies on the assumptions taken in the toy model for simulating mutations corresponding to an evolutionary versus an ecological time-scale, respectively. In this research, we assume that socio-ecological dynamics occur at the short time-scale evolution.

Changes associated with (i) evolutionary time-scales (large time-scale evolution) allowed abrupt changes in FCDN’s topologies, corresponding to changes associated with gains and losses of biological transformation processes (i.e. addition and/or eliminations of links) and/or non-correlated changes in their shares (i.e. random independent changes in link’s weights, i.e. rates of energy transformation). On the other hand, the (ii) short time-scale evolution considers mutations that are more conservative and/or limited, and therefore, relates to changes that occur at ecological time-scales. This algorithm maintains the same network links (i.e. biological transformation processes are fixed), and only allows changes in their weights (i.e. rates of energy processing) around the vicinity of its former value (10% change). The short time-scale evolution then becomes a process that only allows ‘small’ tweaks in links weights for the network. Here, we assume that socio-ecological dynamics occur at the short time-scale, and therefore, we only allow conservative changes for studying the adjustment that greedy strategies use for adapting the system to their own needs.

We use the large time-scale evolution algorithm for adjusting the initialized FCDN’s networks to expected configurations that comply with topologies dictated by the different goal functions (figure 2). Once such evolutionary adjustment is achieved, we posit that the system is now adapted in accordance with the goal function, resulting in naturally evolved network ensembles. We then submit these adjusted network ensembles to the short time-scale evolutionary process, which we assumed as the most suitable time-scale for testing the effects of greedy evolutionary outcomes. In this comparison, the same network ensembles are submitted to both a (i) control evolution, where the optimization of the former goal function continues (but only through conservative mutations in network topologies); and also to a (ii) greedy evolution, where the goal function changes and now optimizes what is required by greedy scenarios (explained below).

#### (ii) Greedy scenarios

Greedy scenarios resemble a situation in which an energy consuming strategy arises within the system (e.g. human societal development) and becomes capable of driving the whole system towards the maximization of their specific requirements, without necessarily complying to the former (systemic) evolutionary dynamic. We claim that these greedy processes can resemble some strategies used by modern human societies at biospheric scales, especially those developed within a socio-metabolic framework (see also Dorninger *et al.* [20] and Lenton *et al.* [51], for mechanisms for obtaining greater energy from socio-ecosystems dynamics).

For instance, modern human societies have greatly increased the energy that flows towards livestock production, with current biomasses surpassing those achieved by wild mammals and even humans [52]. This process can be interpreted as a digression from the regular energy flow dynamics within ecosystems, which now maximizes the energy that flows towards specific forms (e.g. the core node energy forms that would be related to livestock) and could be represented in the toy model as increases in energy flow at those particular core nodes (i.e. greedy nodes). Other similar socio-metabolic dynamics could relate to increases in the rate of processing of certain energy forms, such as burning oil, gas or coal. In regular ecological dynamics, those energy forms have slow processing dynamics—they are generated at geological time-scales, and are rarely processed by known living entities but humans at ecological time-scales. However, humans have powered the construction of their modern societies by processing those extra-metabolic energy sources [5,15,17–19], thereby increasing the energy that flows as byproducts (e.g. CO_2_, methane, etc.). These processes could also be modelled as increases of energy flows at particular greedy nodes. Moreover, humans can also enhance cyclic processes of natural systems for their advantage. For instance, using the same livestock production example, this process requires great amounts of animal feed. Sometimes human practices use livestock manure for feed nutrition and growth, creating a positive feedback between the processes. However, other agricultural practices involve the addition of synthetic fertilizers, which depend on a highly energetic demanding process (the Haber–Bosch process). If the energy used for this energy conversion process is obtained through biomass combustion, an additional cyclic pathway emerges: humans employ vegetative biomass to accelerate the production of nutrients, which are subsequently used to enhance vegetative biomass production. These cyclic energy processes could also be modelled in the toy model by focusing on increasing the energy that cycles through particular core nodes.

We do not aim to evaluate or parametrize the model for detailed societal energy demands in this research. Instead, we focus on executing simulated experiments that forces the increment in energy flow at specific energy forms (i.e. specific nodes) and/or energy cycles (i.e. specific cycles) and compare their systemic effect respect to natural dynamics. This analysis could guide the best energy appropriation strategies that do not compromise system’s evolution. The specific greedy evolution scenarios analysed were:

(i) *local* energy flow appropriation at specific greedy nodes—i.e. enhances of certain energy forms ($\sum_{i\in \mathrm{Greed}}X_{i}^{*}$). This would resemble the development of a greedy strategy that focuses in increasing the energy flowing at some specific energy forms in the balanced system (e.g. livestock production or extra-metabolic energy byproducts), and therefore, focus at greedy nodes for selecting those increments (i.e. local scale). Here, greedy goal functions can (a) select any network change that increases the *absolute flow* in the set of selected greedy nodes; or they could (b) select changes that only increase the *proportional flow* within the selected greedy nodes (compared to the rest of the network). The former selection strategy allows synergies to arise within the system (increases in energy flow within specific greedy nodes can increase the growth of the total system), but this synergy is diminished in the latter situation, as changes are only selected when they can actually sequester proportionally more energy from the system towards the desired energy forms; and
(ii) *autopoietic* appropriation of energy flow by specific network cycles—i.e. enhances in the ‘Finn per node’ of greedy nodes ($\sum_{i\in \mathrm{Greed}}{\mathrm{Finn}}_{i}^{*}$) (corresponding to enhances in their recycling processes). This would resemble the development of a greedy strategy that focuses in increasing energy fluxes through specific cycles (e.g. increasing livestock production by using manure to enhance feed production to feed the livestock). Therefore, this greedy strategy focuses at ‘autopoetic’ scales for selecting the energy increments, which is greater than the local (node) scale, but smaller than the global (network) scale. Once again, greedy goal functions associated to this situation could be (a) *absolute Finn*, or (b) *proportional Finn* with respect to the rest of the network.

### (c) Simulations

We start initializing FCDN’s network ensembles of 30, 50, 70 and 100 core nodes (100 replicates), and subject them to the large time-scale mutation algorithm for 500 evolutionary iterations according to each of the goal functions described (TST, ASC, AMI, EDiff; figure 2). Network connectance is maintained roughly constant in time, at a density of approximately 20% of possible links, which correspond to percentages usually seen in natural systems [53,54]. This is achieved by maintaining the same probability for loosing and/or creating links in the mutation algorithm. Recall that we use this initial procedure for adjusting initialized FCDN’s networks to expected configurations that comply with topologies dictated by the different goal functions, resembling a ‘natural history evolution’ of the system at evolutionary time-scales.

These naturally evolved network ensembles are now submitted to (i) a *control evolution experiment*, where the network continues evolving with the former systemic goal function, and a (ii) *greedy evolution experiment*, where 10%, 30% and 50% of core nodes become greedy according to the four different greedy scenarios described above (figure 2). Recall that for this second evolutionary process, we use the short time-scale mutation algorithm, and therefore, network links are fixed and changes in their weights correspond to 10% of their former value. We let these scenarios evolve for another 5000 iterations, which we confirmed as enough time for the evolving scenarios to stabilize their dynamics (see the electronic supplementary material). We quantify the percentage (proportion) of networks that, when evolved by a greedy experiment per goal function, surpass the performance and scarcity tolerance of its correspondent control evolution experiment.

## 3. Results and discussion

Correlation analyses between goal functions showed that TST and ASC are complementary in our toy model (see the electronic supplementary material for patterns and discussion). This is an interesting outcome, as controversies about their complementarity have been raised in the literature before (e.g. [39]). We will not address those controversies, and will only assume that they are equivalent for this research. Therefore, *we will only address the goal functions of TST, AMI and EDiff in the coming results.*

We compare the (i) *scarcity tolerance* and (ii) *performance* between control versus greedy experiments (i.e. absolute flow, proportion flow, absolute Finn and proportion Finn). We quantify the percentage of greedy evolved networks that surpass in scarcity tolerance and/or performance its correspondent control evolution experiment. These comparisons were made by normalizing their differences with respect to their absolute sum. Therefore, differences in scarcity tolerances are calculated as *b*_diff_ = ((*b*_control_ − *b*_greedy_))/((|*b*_control_| + |*b*_greedy_|)), and differences in performance as goal_diff_ = ((goal_control_ − goal_greedy_))/((|goal_control_| + |goal_greedy_|)) with outcomes between ( − 1, 1). Notice that this normalization is a ratio and not a percentage; and that negative differences group those networks where the greedy experiment surpassed the values of the control experiments, while positive values group the networks were the control experiments surpass the values of the greedy experiment.

### (a) Scarcity tolerance comparison

**Figure 3.**
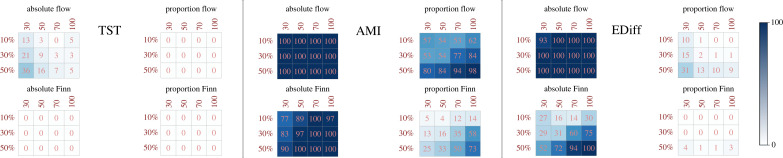
Proportion of greedy network experiments that surpassed the scarcity tolerance of the control evolution scenario, according to each goal function. Columns represent the size of the ensemble (30, 50, 70 and 100 core nodes sizes) and rows represent the percentage of nodes that became greedy (10%, 30%, 50%). Colour bar represents the percentages of greedy networks surpassing the scarcity tolerance achieved by the control evolution, from zero percentages (white) to 100% percentages (dark blue).

Figure 3 shows the proportion of networks from the greedy experiments that surpassed in scarcity tolerance its corresponding control experiment. We can tell that these responses are varied. Specifically, in the TST goal function comparison (figure 3), all greedy experiments perform poorly compared to the control evolution: only some greedy networks selecting for increases in the energy flow of greedy core nodes (absolute flow) can surpass in scarcity tolerance the TST control evolution. The maximum percentage of greedy networks that surpass the control evolution for this scenario is 36%, occurring for the experiment that uses ensembles of 30 core nodes and converts 50% of them as greedy nodes. Moreover, when greedy experiments surpass in scarcity tolerance, the ranges of their difference distributions are low: it is seldom that their difference becomes greater than 0.2 (see the electronic supplementary material).

On the contrary, in the AMI goal function comparison (figure 3), most of the greedy experiments exhibit greater scarcity tolerances than the corresponding control experiment, and their ranges tend to be much higher (the majority of experiments show differences over 0.5 of range; see the electronic supplementary material). In this case, only the greedy experiment that selects for proportional increases in the Finn index of greedy core nodes (proportional Finn) do not greatly exceed AMI control evolution scarcity tolerance outcomes (but shows a maximum of 73% greedy networks that surpasses the scarcity tolerance of the control experiment when using network ensembles of 100 core nodes that convert 50% of them into greedy nodes). Finally, the comparison with EDiff goal function (figure 3) exhibits mixed results, with some greedy experiments completely outperforming the EDiff control evolution scarcity tolerance, such as those selecting for greater absolute flows (where the minimum percentage of greedy networks surpassing control evolution scarcity tolerance was 93% and their gains tended to be over 0.5 points of increases; see the electronic supplementary material); but other greedy experiments cannot surpass the EDiff control evolution, such as the one that selects for greater proportion Finn (where the maximum percentage of greedy networks surpassing the control experiments occur at 4%, with the maximum range of difference at 0.37; see the electronic supplementary material).

The nature of the greedy experiments developed can explain in part why they can surpass so often the scarcity tolerance for AMI and EDiff goal functions. Both AMI and EDiff goal functions stand as orientors that can indeed increase the scarcity tolerance *b* of the whole network when driving the evolution (see the electronic supplementary material); however, the rate of such increases occur slower than the rates at which TST can increase the network’s scarcity tolerance (see the electronic supplementary material). Because all greedy experiments were developed by algorithms that directly or indirectly increase the TST of the network (see the electronic supplementary material), this can explain why so many greedy experiments achieve greater tolerances compared with these goal functions. Moreover, because the short time-scale mutation algorithm does not allow the creation and/or eliminations of new links, the evolution of both AMI and EDiff become more limited than TST, limiting the evolution of network tolerances. The development of other greedy experiments focused specifically at affecting AMI or EDiff indexes are encouraged for properly assessing these comparisons.

Figure 3 also highlights that greedy experiments increasing the energy flow or Finn index per node in an *absolute manner* result in higher percentages of improved scarcity tolerances compared to those experiments that increase those metrics in a *proportional manner*, regardless of the goal function. This pattern suggests that the feasibility of greedy networks for surpassing control evolution scenarios is augmented when synergies of greedy behaviours on certain network components or nodes are allowed to occur with the rest of the system. When these synergies are diminished (this is the role of the proportional evolution scenario experiments), the feasibility to surpass control scarcity tolerances is abruptly reduced in most of the cases.

We also find that the greedy experiments which focus on greater energy flow rather than increases in Finn index per node achieve greater scarcity tolerances than their respective control scenarios. This is not so intuitive, because we would have expected that specifically enhancing system’s cycling would surpass the tolerances achieved than just increasing specific energy flows in a node. As an increase in the Finn index per node is associated with a greater level of cycling within certain system cycles (see the electronic supplementary material), we expected that a selection algorithm which prioritizes this cycling process, compared to one that does not focus on that property, would result in a higher percentage of networks with improved tolerance. However, remember that selection of greedy nodes was made under a random scenario, and therefore, not necessarily nodes with high Finn index could have been selected. Moreover, the short time-scale mutation algorithm also limits Finn index dynamics, precluding the effect of Finn index enhances. For instance, if one node with low Finn index is selected to become greedy, the space parameter that the network has for increasing its Finn index is more limited than the space parameter that such experiment would have for selecting increases in energy flow in that same node. Selection algorithms allowing changes in links topologies could change the pattern.

As expected, greedy experiments that convert greater percentages of core nodes (i.e. 50% over 10%) show greater percentages of networks capable of surpassing the scarcity tolerances achieved by the control experiment, irrespective of the goal function. This happens because greater percentages of greedy nodes in the network increase the effect of greedy strategies within the network. The size of the networks also plays a role, although it changes with respect to the goal function: for TST, and specifically for the greedy experiment that selects for greater absolute energy flows at greedy nodes (the only experiment where greedy scenarios could surpass the tolerances of the control experiment), greater network sizes showed lesser percentages of greedy networks surpassing the control experiment’s scarcity tolerances. However, for AMI, the pattern reverses: here, greater network sizes show greater percentages of greedy networks surpassing the tolerances achieved by the control experiment for all greedy experiments. For EDiff, the role of the network size is mixed: greedy experiments that increase both the energy flow and Finn index per node in an *absolute manner* achieve greater percentages of greedy networks that surpass the control experiment with increasing network size; but those greedy experiments that increase both indexes in a *proportional manner* show a negative relationship between the percentages of greedy networks that can surpass the scarcity tolerances of the control experiment.

### (b) Performance comparison

**Figure 4.**
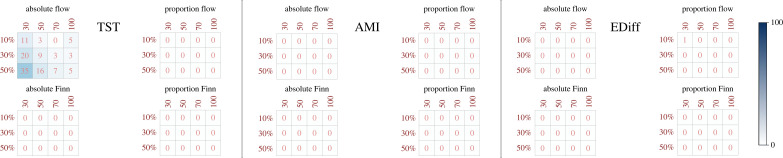
Percentage of networks from the four different greedy evolution scenarios that surpassed the goal orientor performance of the control evolution scenario, according to each goal orientor. Columns represent the size of the ensemble (30, 50, 70 and 100 core nodes sizes) and rows represent the percentage of nodes that became greedy (10%, 30%, 50%). Colour bar represents the percentages of greedy networks surpassing the performance achieved by the control evolution, from zero percentages (white) to 100% percentages (dark blue).

Figure 4 shows the same analysis performed for scarcity tolerances, but now focusing on the performance of greedy experiments versus control experiments. Therefore, for each goal function, we quantify the percentage of greedy networks per greedy experiment that surpassed the value reached by the goal function compared to the control evolution experiment.

From first sight, figure 4 shows that almost no greedy experiment for any goal function can surpass the performance of the control evolution. Once again, this pattern is somehow expected for the AMI and EDiff goal functions, as the greedy experiments developed in this research are not intimately associated with enhancers for these orientors (see the electronic supplementary material). This is not necessarily the case for the TST goal function: all greedy experiments associate to increases in the TST of the system, especially the greedy experiment that increases the absolute energy flow of greedy nodes (see the electronic supplementary material). However, only some percentages of greedy networks could surpass the performance of the control experiment when selecting for increases in the absolute flow of greedy nodes (figure 4), and their ranges are always smaller than the ranges achieved by the TST control experiment (see the electronic supplementary material). What is striking about these numbers is that they are very similar to the percentages shown in the scarcity tolerance comparison (compare with TST values in figure 3). We study this relationship further.

**Figure 5.**
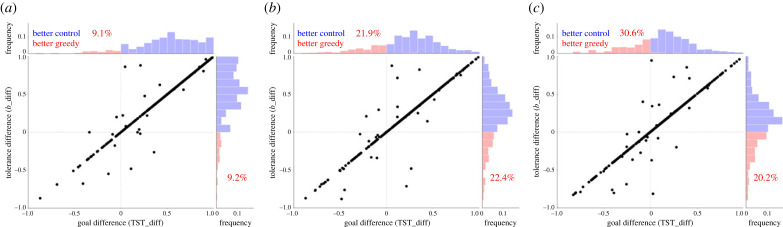
Analysis of the differences in scarcity tolerance and performance between systemic TST control evolution versus increases in absolute energy flow in greedy nodes, using 1000 replicates of 30 core nodes FCDN’s ensembles. Dotted lines separate the positive from the negative differences, with negative values representing those networks where the greedy scenario surpassed the outcomes of the control evolution (‘better greedy’, in red). The main plot is supported by histograms of the point projections towards their corresponding axis, with percentages shown in red text. (*a*) 10% greedy nodes, (*b*) 30% greedy nodes, (*c*) 50% greedy nodes.

For that, we re-ran the simulation that compares the evolution of increasing systemic TST (control experiment) versus increases in absolute energy flow in greedy nodes, using 1000 replicates of 30 core nodes FCDN’s ensembles. This analysis consolidated scarcity tolerance and performance trends determined with the 100 replicates experiments (compare percentages shown for TST in figures 3 and 4 with those shown in red in figure 5 and the electronic supplementary material). Moreover, this analysis confirmed that those greedy networks achieving greater scarcity tolerances than the control experiment are the same networks that also surpass it in performance (figure 5). Furthermore, this trend happens in a linear positive relationship (for all greedy nodes percentages’ conversions), suggesting the existence of a space parameter that allows greedy strategies to increase the energy flow at specific network components and with this, outperforming the systemic power achieved by natural evolution. However, the feasibility of such an outcome depends on whether the greedy strategy is also capable of enhancing the system’s tolerances in a similar manner.

However, no unique network property seems to explain the former pattern (see the electronic supplementary material). First, we studied whether greedy networks whose greedy nodes comprised greater percentages of the total network’s Finn index were responsible for that distribution. In other words: we tested whether the reason that greedy experiments can surpass the TST control evolution relates to the fact that those networks contain greedy nodes that monopolize the cycles in the networks, i.e. contain high percentages of the total Finn index, dominating the FCDN’s dynamic. However, this analysis did not show the expected positive trend, and the majority of networks that contain the greatest percentages of Finn index grouped within greedy nodes did not show greater scarcity tolerance nor performance than their control evolution counterpart (see the electronic supplementary material). We then studied whether the number of links that greedy nodes have towards the output could explain the pattern. We would expect that greedy networks’ scarcity tolerance and performance are negatively affected whenever selected greedy nodes are directly connected to the output nodes, precluding further synergies with the rest of the network. The pattern for this analysis is more clear and goes as expected: greedy networks tend to exhibit higher scarcity tolerances when the greedy nodes have fewer link connections towards the output node, regardless of the proportion of greedy nodes within the system. However, once again, this metric appears as necessary but not sufficient for surpassing the control experiments (see the electronic supplementary material). However, the trend suggests that greedy strategies focusing on greater energy processing can enhance system’s performance and tolerances when their effect recycle through the system (creating synergies) instead of loosing such capacity as byproducts.

## 4. Conclusion and future research

In this research, we describe an energy-based flow network toy model of open natural systems and developed *in silico* experiments for comparing the scarcity tolerance and performance outcomes of systemic versus biased evolutionary dynamics. Systemic evolution resembled natural evolutionary dynamics, and three main indexes were finally used as goal functions or orientors: (i) TST, which associates with system’s growth through power; (ii) AMI, which associates to system’s development; and (iii) EDiff, which is a new proposed index that quantifies the exported entropy by the system. On another hand, biased evolutionary dynamics focused on energy appropriation at particular network substructures, resembling greedy strategies that do not necessarily comply to systemic dynamics. We tested four different scenarios of greedy strategies, emphasizing the absolute or proportional energy accumulation at local (node) or autopoetic (cycles) scales in the network. We aim to determine the flexibility that biased evolutionary dynamics have for matching or surpassing the scarcity tolerance and performance of natural evolution.

The nature of the greedy scenarios developed were closely related to increases in TST and therefore, we emphasized this goal function comparison. This focus seems adequate, since both ecology [22,26,27,55] and societal systems [56,57] have theories that focus on increases in system’s power. The results showed that greedy scenarios rarely surpassed the scarcity tolerance and performance achieved by the control evolution experiment: some cases were only feasible when increasing the absolute energy flow at local (node) scale. Here, the maximum percentage of greedy networks capable of surpassing the tolerance and performance achieved by the control evolution was around one-third (approx. 35%), occurring for the experiment that used the smallest FCDN’s sizes (30 core nodes FCDN’s ensembles) and the greatest percentage of greedy nodes conversion (50% of total core nodes). These outcomes point out the difficulties that greedy strategies have for surpassing natural evolutionary dynamics.

Interestingly though, is the linear relationship we found between scarcity tolerance and performance shown by those greedy networks that outperform the TST control evolution. Therefore, greedy networks achieving greater power (i.e. TST performance) than the control experiment are also the same networks that surpass the control experiment in scarcity tolerance in a similar proportion. This suggests the existence of a space parameter where greedy strategies focusing at local increases of energy flow can outperform the outcomes of systemic natural evolution. However, no clear network property seems to characterize those greedy networks. A mild relationship was found between the number of links that greedy nodes have towards the output and their capacity to outpass the TST control evolution. We found that whenever greedy nodes have fewer link connections towards the output node, greedy networks can surpass the control evolution. However, this metric appears as necessary but not sufficient for surpassing the control experiments. This is an interesting relationship, as many of the modern human activities do not emphasize recycling dynamics and mostly create dissipative byproducts. Therefore, knowing that such dynamics negatively affect system’s tolerance and performance suggest care in their use.

Finally, it is worth mentioning that other mutation and/or selection algorithms could be developed for continued testing of the effects that biased evolutionary dynamics can have upon the system’s scarcity tolerance, performance and other metrics not discussed in this research. As an example, it could be interesting to study the effects of a combination of goal functions as the evolutionary driver, instead of focusing on a single goal function. Also, the effect that gains and/or losses of links can create when assumed as an evolving trait in the greedy evolutionary strategy, the control evolution scenario, or both; as well as modelling other biased evolutionary strategies that could adjust to desired scarcity tolerances and well-performed configurations (and other metrics). Lastly, there were some analyses that we only developed at an exploratory level in the electronic supplementary material, and that are worth of further research. For instance, the relationships between our metric of mutual information (AMI) and the other goals functions was only reported but not analysed in depth.

## Data Availability

All code and data can be openly accessed from the GitHub digital repository: https://github.com/vanewi/flowneṫevolution. The data are provided in the electronic supplementary material [58].

## References

[1] Schrödinger E. 1944 What is Life? The physical aspect of the living cell. Cambridge, UK: Cambridge University Press.

[2] Morowitz H, Smith E. 2007 Energy flow and the organization of life. Complexity **13**, 51-59. (10.1002/cplx.20191)

[3] Brown JH, Gillooly JF, Allen AP, Savage VM, West GB. 2004 Toward a metabolic theory of ecology. Ecology **85**, 1771-1789. (10.1890/03-9000)

[4] Ellis EC. 2015 Ecology in an anthropogenic biosphere. Ecol. Monogr. **85**, 287-331. (10.1890/14-2274.1)

[5] Schramski JR, Gattie DK, Brown JH. 2015 Human domination of the biosphere: rapid discharge of the earth-space battery foretells the future of humankind. Proc. Natl Acad. Sci. USA **112**, 201508353. (10.1073/pnas.1508353112) PMC4534254 26178196

[6] Lenton TM, Pichler PP, Weisz H. 2016 Revolutions in energy input and material cycling in Earth history and human history. Earth Syst. Dyn. **7**, 353-370. (doi:10.5194/esd-7-353-2016)

[7] Judson OP. 2017 The energy expansions of evolution. Nature Ecol. Evol. **1**, 1-9. (10.1038/s41559-017-0138) 28812646

[8] Crutzen PJ. 2002 Geology of mankind. Nature **415**, 23-23. (10.1038/415023a) 11780095

[9] Rockström J et al. 2009 Planetary boundaries: exploring the safe operating space for humanity. Ecol. Soc. **14**, 32. (10.5751/ES-03180-140232)

[10] Barnosky AD et al. 2012 Approaching a state shift in Earth’s biosphere. Nature **486**, 52-58. (10.1038/nature11018) 22678279

[11] Steffen W et al. 2015 Planetary boundaries: guiding human development on a changing planet. Science **347**, 1259855. (10.1126/science.1259855) 25592418

[12] Lade SJ et al. 2020 Human impacts on planetary boundaries amplified by Earth system interactions. Nature Sustainab. **3**, 119-128. (10.1038/s41893-019-0454-4)

[13] Nielsen SN, Fath B, Bastianoni S, Marques JC, Muller F, Patten BD, Ulanowicz RE, Tiezzi E. 2019 A new ecology: systems perspective. Amsterdam, The Netherlands: Elsevier. Google-Books-ID: ITSsDwAAQBAJ.

[14] Schramski JR, Dell AI, Grady JM, Sibly RM, Brown JH. 2015 Metabolic theory predicts whole-ecosystem properties. Proc. Natl Acad. Sci. USA **112**, 2617-2622. (10.1073/pnas.1423502112) 25624499 PMC4345554

[15] Williams M, Zalasiewicz J, Haff P, Schwägerl C, Barnosky AD, Ellis EC. 2015 The Anthropocene biosphere. Anthropocene Rev. **2**, 196-219. (10.1177/2053019615591020)

[16] Ellis EC. 2023 The Anthropocene condition: evolving through social-ecological transformations. Phil. Trans. R. Soc. B **378**, 20220255. (10.1098/rstb.2022.0255) PMC10645118 37952626

[17] Burger JR, Weinberger VP, Marquet PA. 2017 Extra-metabolic energy use and the rise in human hyper-density. Sci. Rep. **7**, 43869. (10.1038/srep43869) 28252010 PMC5333137

[18] Weinberger VP, Quiñinao C, Marquet PA. 2017 Innovation and the growth of human population. Phil. Trans. R. Soc. B **372**, 20160415. (10.1098/rstb.2016.0415) 29061888 PMC5665803

[19] Snyder BF. 2020 The genetic and cultural evolution of unsustainability. Sustainability Sci. **15**, 1087-1099. (10.1007/s11625-020-00803-z) PMC7133775 32292525

[20] Dorninger C, Menéndez LP, Caniglia G. 2023 Social-ecological niche construction for sustainability: understanding destructive processes and exploring regenerative potentials. Phil. Trans. R. Soc. B **378**, 20220431. (10.1098/rstb.2022.0431) PMC10645119 37952625

[21] Lotka AJ. 1922 Contribution to the energetics of evolution. Proc. Natl Acad. Sci. USA **8**, 147-151. (10.1073/pnas.8.6.147) 16576642 PMC1085052

[22] Odum HT, Pinkerton RC. 1955 Time’s speed regulator: the optimum efficiency for maximum power output in physical and biological systems. Am. Sci. **43**, 331-343.

[23] Solée RV, Valverde S, Casals MR, Kauffman SA, Farmer D, Eldredge N. 2013 The evolutionary ecology of technological innovations. Complexity **18**, 15-27. (10.1002/cplx.21436)

[24] Levin SA. 1998 Ecosystems and the biosphere as complex adaptive systems. Ecosystems **1**, 431-436. (10.1007/s100219900037)

[25] Odum HT. 1988 Self-organization, transformity, and information. Science **242**, 1132-1139. (10.1126/science.242.4882.1132) 17799729

[26] Nielsen SN, Müller F, Marques JC, Bastianoni S, Jørgensen SE. 2020 Thermodynamics in ecology–an introductory review. Entropy **22**, 820. (10.3390/e22080820) 33286591 PMC7517404

[27] Kleidon A. 2022 Working at the limit: a review of thermodynamics and optimality of the Earth system. Earth Syst. Dyn. Discuss. **2022**, 1-46. (10.5194/esd-2022-38)

[28] Nielsen SN, Ulanowicz RE. 2000 On the consistency between thermodynamical and network approaches to ecosystems. Ecol. Modell. **132**, 23-31. (10.1016/S0304-3800(00)00302-1)

[29] Fath BD, Patten BC, Choi JS. 2001 Complementarity of ecological goal functions. J. Theor. Biol. **208**, 493-506. (10.1006/jtbi.2000.2234) 11222052

[30] Ulanowicz RE, Jørgensen SE, Fath BD. 2006 Exergy, information and aggradation: an ecosystems reconciliation. Ecol. Modell. **198**, 520-524. (10.1016/j.ecolmodel.2006.06.004)

[31] Jain S, Krishna S. 2001 A model for the emergence of cooperation, interdependence, and structure in evolving networks. Proc. Natl Acad. Sci. USA **98**, 543-547. (10.1073/pnas.98.2.543) 11149953 PMC14623

[32] Ulanowicz RE. 1997 Ecology, the ascendent perspective. New York, NY: Columbia University Press.

[33] Lenton TM, Kohler TA, Marquet PA, Boyle RA, Crucifix M, Wilkinson DM, Scheffer M. 2021 Survival of the systems. Trends Ecol. Evol. **36**, 333-344. (10.1016/j.tree.2020.12.003) 33414020

[34] Fath BD, Patten BC. 1999 Review of the foundations of network environ analysis. Ecosystems **2**, 167-179. (10.1007/s100219900067)

[35] Braakman R, Follows MJ, Chisholm SW. 2017 Metabolic evolution and the self-organization of ecosystems. Proc. Natl Acad. Sci. USA **114**, E3091-E3100. (10.1073/pnas.1619573114) 28348231 PMC5393222

[36] Odum EP. 1969 The strategy of ecosystem development. Science **164**, 262-270. (10.1126/science.164.3877.262) 5776636

[37] Fath BD et al. 2019 Ecological network analysis metrics: the need for an entire ecosystem approach in management and policy. Ocean Coast. Manage. **174**, 1-14. (10.1016/j.ocecoaman.2019.03.007)

[38] Latham LG, Scully EP. 2002 Quantifying constraint to assess development in ecological networks. Ecol. Modell. **154**, 25-44. (10.1016/S0304-3800(02)00032-7)

[39] Latham LG, Scully EP. 2004 Network optimization model implies strength of average mutual information in ascendency. Ecol. Modell. **179**, 373-392. (10.1016/j.ecolmodel.2004.04.017)

[40] Finn JT. 1976 Measures of ecosystem structure and function derived from analysis of flows. J. Theor. Biol. **56**, 363-380. (10.1016/S0022-5193(76)80080-X) 944838

[41] Allesina S, Ulanowicz RE. 2004 Cycling in ecological networks: Finn’s index revisited. Comput. Biol. Chem. **28**, 227-233. (10.1016/j.compbiolchem.2004.04.002) 15261153

[42] Ulanowicz RE. 2004 On the nature of ecodynamics. Ecol. Complex. **1**, 341-354. (10.1016/j.ecocom.2004.07.003)

[43] Xu Z, Cheng G, Ulanowicz RE, Song X, Deng X, Zhong F. 2018 The common developmental road: tensions among centripetal and centrifugal dynamics. Nation. Sci. Rev. **5**, 417-426. (10.1093/nsr/nwx033)

[44] Borrett SR, Fath BD, Patten BC. 2007 Functional integration of ecological networks through pathway proliferation. J. Theor. Biol. **245**, 98-111. (10.1016/j.jtbi.2006.09.024) 17084414

[45] Gatti RC, Fath B, Hordijk W, Kauffman S, Ulanowicz R. 2018 Niche emergence as an autocatalytic process in the evolution of ecosystems. J. Theor. Biol. **454**, 110-117. (10.1016/j.jtbi.2018.05.038) 29864429

[46] Pimm SL, Lawton JH. 1977 Number of trophic levels in ecological communities. Nature **268**, 329-331. (10.1038/268329a0)

[47] Neubert MG, Caswell H. 1997 Alternatives to resilience for measuring the responses of ecological systems to perturbations. Ecology **78**, 653-665. (10.1890/0012-9658(1997)078[0653:atrfmt]2.0.co;2)

[48] Müller F, Leupelt M eds. 1998 Eco targets, goal functions, and orientors. Berlin, Germany: Springer.

[49] Patten BC. 1995 Network integration of ecological extremal principles: exergy, emergy, power, ascendency, and indirect effects. Ecol. Modell. **79**, 75-84. (10.1016/0304-3800(94)00037-I)

[50] Jørgensen SE. 1999 State-of-the-art of ecological modelling with emphasis on development of structural dynamic models. Ecol. Modell. **120**, 75-96. (10.1016/S0304-3800(99)00093-9)

[51] Lenton TM, Scheffer M. 2023 Spread of the cycles: a feedback perspective on the Anthropocene. Phil. Trans. R. Soc. B **378**, 20220254. (10.1098/rstb.2022.0254) PMC10645129 37952624

[52] Bar-On YM, Phillips R, Milo R. 2018 The biomass distribution on Earth. Proc. Natl Acad. Sci. USA **115**, 201711842. (10.1073/pnas.1711842115) PMC6016768 29784790

[53] Newman M. 2010 Networks: an introduction. Oxford, UK: Oxford University Press.

[54] Dunne JA, Williams RJ, Martinez ND. 2002 Food-web structure and network theory: the role of connectance and size. Proc. Natl Acad. Sci. USA **99**, 12 917-12 922. (10.1073/pnas.192407699) PMC130560 12235364

[55] Lotka AJ. 1922 Natural selection as a physical principle. Proc. Natl Acad. Sci. USA **8**, 151-154. (10.1073/pnas.8.6.151) 16576643 PMC1085053

[56] Stefan G. 2012 Considerations on the theory of economic growth and development. Procedia - Soc. Behav. Sci. **62**, 280-284. (10.1016/j.sbspro.2012.09.045)

[57] Huang J, Ulanowicz RE. 2014 Ecological network analysis for economic systems: growth and development and implications for sustainable development. PLoS ONE **9**, e100923. (10.1371/journal.pone.0100923) 24979465 PMC4076221

[58] Weinberger VP, Zalaquett N, Abades S. 2023 How greedy is too greedy? A network toy model for evaluating the sustainability of biased evolutionary dynamics. *Figshare*. (10.6084/m9.figshare.c.6858669) PMC10645075 37952630

